# Intestinal Pseudo-Obstruction as an Unusual Gastrointestinal Presentation in Pediatric Human Immunodeficiency Virus Infection

**Published:** 2014-01

**Authors:** Mozhgan Zahmatkeshan, Mahmood Haghighat, Mohammad Hadi Imanieh

**Affiliations:** 1 Department of Pediatric Gastroenterology, School of Medicine, Shiraz University of Medical Sciences, Shiraz, Iran

**Keywords:** HIV, Intestinal pseudo-obstruction, Abdominal pain, Children

## Abstract

Intestinal pseudo-obstruction is a condition in which the intestine’s ability to push food through is reduced. It often leads to the dilation of the various parts of the bowel. It can be idiopathic or inherited from a parent, or caused by another disease. We report a rare case of human immunodeficiency virus (HIV) infection in a 3-year-old boy who referred with acute abdominal pain, and was later diagnosed as having intestinal pseudo-obstruction caused by HIV.

The underlying causes of intestinal pseudo-obstruction should be taken into account. HIV induced pseudo-obstruction may be considered in the differential diagnosis of pediatric intestinal pseudo-obstruction in order to provide a timely diagnosis and optimal care of children with HIV.

## Introduction

Pediatric human immunodeficiency virus (HIV) infection is an important world health problem, with its prevalence increasing at an alarming rate. 2500 young people were infected per day In 2009.^1^ In most pediatric cases, HIV virus is usually transmitted from an HIV positive mother to the child during pregnancy, delivery, or breast feeding.^2^ Moreover, in children infected with HIV, immune system dysfunction and its related complications progress more rapidly compared with adults.^3^

The clinical manifestations of HIV infection in infants and children are varied and include lymphadenopathy, hepatosplenomegaly, failure to thrive, oral candidiasis, chronic parotitis, chronic cough, generalized dermatitis, Pneumocystis jirovecii pneumonia, recurrent bacterial infections, and wasting syndrome.^4^

Gastrointestinal manifestations such as diarrhea, jaundice, dysphasia, abdominal pain, and gastrointestinal bleeding are the common presentations of HIV/AIDS in children.^5^ Most of these symptoms are related to giardiasis, candidiasis or infections with cytomegalovirus, or mycobacteria.^4^^,^^6^ Intestinal obstruction caused by the varicella–zoster virus,^7^ mycobacterium avium intracellular, cytomegalovirus, Cryptosporidium parvum, lymphoma, and Kaposi’s sarcoma^8^ may be seen in patients with HIV.

However, intestinal pseudo-obstruction is not a usual finding in HIV disease. Some studies have reported intestinal pseudo-obstruction as a complication of strongyloides stercoralis,^9^ or due to the side effects of medications used for HIV treatment. Herein we describe a patient infected with HIV who presented with unusual intestinal pseudo-obstruction.

## Case Report

The patient was a 3-year-old boy who referred to the Pediatric Gastroenterology Ward with abdominal distension, diarrhea, and failure to thrive. The child had had these complications since he was 6 months old. He had had several hospital admissions due to bouts of acute abdominal pain, consistent with intestinal obstruction. 

Abdominal radiography showed dilated bowel loops, in favor of intestinal obstruction. Abdominal sonography and upper gastrointestinal series were normal. Barium enema showed a dilated long segment of the sigmoid colon, suggestive of Hirschsprung’s disease. So, laparotomy was performed to differentiate between intestinal obstruction and probable Hirschsprung’s disease. No evidence of obstruction was observed in laparotomy. Therefore, full thickness biopsy sample of the sigmoid colon was taken. The biopsy sample showed normal ganglion cells. Anorectal manometry was done and showed normal rectoanal inhibitory reflex (RAIR) without any evidence of Hirschsprung’s disease.

Upper gastrointestinal endoscopy was done, and a duodenal biopsy sample was also taken. It showed villous atrophy and cyclospora infestation without any evidence of celiac disease. Medical treatment for cyclospora was started with Trimethoprim/Sulfamethoxazole. However, diarrhea and abdominal distension continued. Moreover, stool examination was positive for cryptosporidium, and hence, Nitazoxanide was started for the patient. To rule out the immune system dysfunction, immunological examinations were performed, which showed normal CD4^+^, CD8^+^, and NK cells but CH4+/CH8+ was 0.05 (normal: 0.9-1.9). The enzyme-linked immunosorbent assay (ELISA) test was positive for HIV. Further investigation using the western blot approved the diagnosis of HIV in the child and his parents. Since intestinal pseudo-obstruction is not usually found in patients with HIV, the medical team was misguided in the diagnosis of HIV for this patient.

A complete medical history taking and physical examination was done for the parents. The father was known to be addicted to inhalational opium and a tattoo was found on his arm. 

## Discussion

Intestinal obstruction is a common cause of acute abdominal pain in children. Although intestinal obstruction may be seen in patients with HIV either before treatment or as a consequence of medical therapy especially after treatment with Ritonavir and Lopinavir, intestinal pseudo-obstruction is not a common manifestation of HIV disease. 

In our patient, cryptosporidium was found in the stool examination. Imaging studies were performed and led to the diagnosis of intestinal pseudo-obstruction. The patient’s condition was improved after treating the cryptosporidium infection. It is probable that the gastrointestinal pseudo-obstruction had arisen from cryptosporidium infection.

Cryptosporidium is usually found in patients with HIV and is often asymptomatic. It may also cause diarrhea and abdominal colics. A previous report by Aeri Moon et al.^10^ demonstrated that a patient with HIV infection presented with repeated vomiting and chronic diarrhea unresponsive to medications, including metronidazole, azithromycin, ondansetron hydrochloride, and intravenous octreotide. Upper gastrointestinal series and esophagogastroduodenoscopy with tissue biopsy showed cryptosporidial infection as the cause of gastric outlet obstruction. Intestinal pseudo-obstruction is not a usual finding. In our patient, however, there was no proven mechanical obstruction and it seems that the clinical manifestations of the gastrointestinal tract were due to intestinal pseudo-obstruction. The mechanism of diarrhea in cryptosporidium infection is poorly understood. But cryptosporidium seems to induce a rearrangement of the host enterocyte cytoskeleton, reducing the membrane expression of nutrient transporters and causing impaired absorption and osmotic diarrhea. The other gastrointestinal manifestations of cryptosporidial infection are severe, life-threatening enteritis complicated by the biliary tract involvement, severe abdominal cramping, electrolyte imbalance, and severe dehydration.^10^ Whether similar mechanisms are involved in inducing intestinal pseudo-obstruction is not clear. A similar case report by Pui JC^8^ detected the varicella-zoster virus in the muscularis propria layer of the terminal ileum in an HIV-positive patient who presented with the signs and symptoms of intestinal pseudo-obstruction. Pui JC postulated that the varicella-zoster virus infection can damage the neuronal plexus in the muscularis propria layer, thereby mimicking the manifestations of intestinal pseudo-obstruction.

Children with HIV have a higher rate of hospitalization compared with healthy children.^4^ Therefore, the diagnosis of HIV must be considered in pediatric patients, especially when all usual diagnostic approaches have failed to provide explanations for the patients’ signs and symptoms. We ruled out other usual causes of intestinal obstruction in children, including intestinal malrotation, intussusception, and Hirschsprung’s disease.

## Conclusion

The child described in this report had attacks of intestinal pseudo-obstruction as the main clinical manifestation of his disease. We suggest that HIV be considered in the differential diagnosis of pediatric intestinal pseudo-obstruction to provide a timely diagnosis and optimal care of children with HIV. 
